# Influence of theoretical and practical artificial intelligence knowledge on the primary school teachers’ sustainable AI integration ability: mediating effects of beliefs and attitudes

**DOI:** 10.3389/fpsyg.2025.1628557

**Published:** 2025-08-05

**Authors:** Aytun Kazmaci, Kemal Cek, Fahriye Altinay, Zehra Altinay, Gokmen Dagli

**Affiliations:** ^1^Faculty of Education, Near East University, Nicosia, Cyprus; ^2^Faculty of Economics and Administrative Sciences, Cyprus International University, Nicosia, Cyprus; ^3^Faculty of Education, University of Kyrenia, Kyrenia, Cyprus

**Keywords:** artificial intelligence, primary education, theoretical AI knowledge, practical AI knowledge, beliefs and attitudes, sustainable AI integration, teachers

## Abstract

Artificial intelligence (AI) has great potential to be integrated into education to improve teaching and learning practices. However, the attainment of effective and sustainable AI tools usage in the primary education of the teachers’ knowledge, beliefs, and attitudes is critical. This study examines the impact of theoretical and practical AI knowledge on the sustainable integration of AI in primary school classrooms, with a focus on the mediating role of teachers’ beliefs and attitudes. A structured questionnaire was used to collect data from 340 primary school teachers in Northern Cyprus. Structural Equation Modeling (SEM) was used to test the relationships between AI knowledge, beliefs and attitudes and sustainable AI integration. Findings indicate that both theoretical and practical AI knowledge indirectly contribute to the integration of AI. Specifically, the beliefs and attitudes of teachers toward AI are a critical mediator that maintains the relationship. The results underscore the importance of training teachers to enhance their understanding of AI, cultivate positive attitudes, and boost their confidence in AI technologies. To offer better strategies that enhance the sustainable integration of AI in primary education, educational policymakers and institutions must consider these psychological factors that need to be addressed. This research contributes to the emerging body of work on AI in education, in general, and the interplay between knowledge, attitudes, and AI adoption, in particular, emphasizing the need for a holistic strategy toward teacher development.

## 1 Introduction

Modern pedagogy has undergone a significant transformation with the advent of artificial intelligence (AI), creating multiple educational options (Luckin et al., 2022). The complete utilization of AI requires teachers to comprehend its pedagogical benefits, which come from AI-based instructional tools (Xu, 2020). Teachers who want to use AI tools effectively in their teaching practices must possess sufficient pedagogical knowledge (Cavalcanti et al., 2021). Teachers who perceive AI tools to deliver practical advantages apply them with improved proficiency to increase student motivation and engagement. Teachers who understand AI well possess the capability to select suitable AI tools for educational applications (Edwards et al., 2018). The educators’ understanding of AI enables them to work with AI-based technologies that deliver customized lessons supported by quick feedback systems (Popenici and Kerr, 2017). Educators’ assessment of their AI tool integration skills in education demonstrates critical importance because technology and teaching expertise mutually determine the success rates of any sustainable technology integration within classrooms (Mishra and Koehler, 2006).

Next-generation pedagogical methods can be achieved through AI-based tools which support both the educational needs of learners and instructors. AI-based tools are a promising educational opportunity to develop learner-centered educational practices (Luan et al., 2020). AI-based tools enhance students’ personalized experiences by fostering a learner-centered teaching environment (Hwang et al., 2020; Luckin and Cukurova, 2019; Shum, 2019). The AI system enables instructors to deliver valid summative and formative evaluations of student knowledge (Celik et al., 2022). Existing research demonstrates how AI-based tools help teachers execute educational process assessments for teaching and developing classroom content (Celik et al., 2022; Zawacki-Richter et al., 2019). Sustainable AI integration refers to the long-term, consistent, and pedagogically meaningful use of artificial intelligence technologies in educational settings. It emphasizes not only the initial adoption of AI tools by teachers but also their continued, adaptive use over time, supported by sufficient knowledge, positive attitudes, institutional backing, and evolving pedagogical strategies (Chiu and Chai, 2020; Tondeur et al., 2017). In the context of primary education, sustainable integration involves embedding AI tools into teaching practices in a way that is contextually appropriate, ethically responsible, and aligned with student learning goals, ensuring enduring benefits rather than short-term or superficial integration (Celik, 2023; Nazaretsky et al., 2022).

The introduction of AI alters numerous societal elements through its ability to automate complex processes across various markets, significantly impacting sustainable progress (Jordan and Mitchell, 2015). The application of AI in education offers transformative prospects to both evaluate traditional educational methods and integrate personalized educational approaches which are sustainably efficient. Most existing AI education research focuses on system development, while neglecting key elements that determine the adoption and persistence of AI technologies in K-12 learning environments (Zawacki-Richter et al., 2019). Researchers have begun to recognize teacher perceptions about sustainable integration of AI as a crucial field of study in the last few years (Chiu and Chai, 2020). The State Council of China reveals in their “Development Plan of New Generation Artificial Intelligence” that artificial intelligence methods will lead to permanent educational innovations (2017). In 2018, the Ministry of Education in China established AI-education demonstration districts to study trustworthy AI-integrated educational approaches that can be applied across various subjects. Long-term deployment success for AI education requires a deeper understanding of teachers’ perceptions toward AI, allowing new strategies to emerge that permanently embed AI teaching methodologies (Luo et al., 2025).

The analysis examines the relationship between primary school teachers’ background knowledge of artificial intelligence and their current ability to integrate AI devices in their educational practice. This investigation reveals that either theoretical or practical knowledge about AI has a significant influence on teachers who continue to use AI educational tools. The research analysis examines whether psychological elements, such as teachers’ beliefs and attitudes, serve as intermediaries between AI understanding and durable AI adoption. This research examines the mediators that influence the interaction between cognitive elements and emotional variables when teachers adopt and sustain the use of AI in educational environments. The research enhances existing educational technology frameworks by independently defining practical and theoretical AI knowledge. The empirical research gathers valuable information about teacher beliefs and attitudes as critical factors that explain the teachers’ ability to integrate AI in primary education sustainably. The research findings supply education decision-makers, primary school leaders, and teacher educators with concrete guidance for developing targeted professional development initiatives. Educational stakeholders must unite theoretical instruction with practical AI experience to help teachers effectively and sustainably integrate such technology. Future research projects utilizing educational technologies should refer to this research design as their systematic evaluation framework.

Despite the increasing focus on AI in education, research addressing the sustainable integration of AI—particularly within primary school contexts—remains limited (Chiu and Chai, 2020; Su and Yang, 2022). Much of the current literature emphasizes either the general adoption of AI tools or technical implementation models (Zawacki-Richter et al., 2019; Chiu et al., 2023), without adequate attention to the long-term, continuous use that reflects sustainable pedagogical transformation. Moreover, studies often overlook the interplay between teachers’ pedagogical readiness and emotional commitment, which are crucial for lasting integration (Ertmer and Ottenbreit-Leftwich, 2010; Tondeur et al., 2017). This gap is especially pressing in primary education, where early teacher practices can significantly shape children’s digital competencies and openness to AI-supported learning (Ali et al., 2019; UNICEF, 2024). Responding to this gap, the present study investigates how theoretical and practical knowledge of AI influences the teachers’ ability to sustainably integrate AI in primary schools, with a particular focus on the mediating effects of teachers’ beliefs and attitudes. By illuminating the psychological and cognitive mechanisms underlying AI integration, the study contributes to the development of evidence-based strategies for teacher training, institutional support, and policy design that promote long-term AI adoption in education.

The remainder of this paper is structured as follows: Section 2 presents the literature review and hypotheses. Section 3 details the methodology. Section 4 outlines the results. Section 5 offers a structured discussion, and Section 6 concludes the study with implications and directions for future research.

## 2 Literature review

### 2.1 AI-integrated education

AI-integrated education is an ongoing interdisciplinary discipline that implements modern AI technologies to transform educational procedures, creating enhanced educational experiences. The use of AI in science teaching alongside learning contexts has gained broad acceptance alongside increased general education interest in AI implications (Chiu et al., 2023). The educational evaluation and assessment of scientific models in educational institutions use machine learning methods as one major category of AI technology. For instance, several studies have demonstrated how machine learning (ML) algorithms can identify students’ science-based activity responses to provide fast and accurate assessment feedback (Haudek and Zhai, 2024; Zhai et al., 2022; Zhai et al., 2020a,b). Additionally, some research studied how intelligent technologies impact traditional instructional methods and student learning experiences in higher education (Chiu et al., 2023; Popenici and Kerr, 2017; Zawacki-Richter et al., 2019).

### 2.2 Theoretical and practical AI knowledge

Educational studies utilized theoretical and practical knowledge to explain the separate effects of these approaches on instructional methods and student learning activities. Systemic principles and conceptual frameworks comprise the theoretical knowledge that people acquire through formal educational programs or structured training settings (Eraut, 2000). Practical knowledge emerges from experiential learning, representing the skills, insights, and competencies that develop through direct practice, real-world interaction, and application (Fenstermacher, 1994). Theoretical knowledge provides educators with fundamental principles that explain instructional decisions, while practical knowledge equips teachers to apply these principles successfully in multiple educational settings (Shulman, 1987). Theoretical knowledge forms the basis for efficient practice, but practical knowledge decisively affects how well and enduringly theory-based methods are implemented (Korthagen et al., 2001). The sustainable integration of educational innovations, such as artificial intelligence technologies, requires an optimal blend of theoretical conceptualization and hands-on ability to create enduring, meaningful results (Celik, 2023; Lee et al., 2024; Mishra and Koehler, 2006).

Expert studies about integrating technology in education separate theoretical and practical knowledge by analyzing their distinct functions and training effects on educational instruction practices. Academic knowledge about technology encompasses the abstract theoretical aspects of tool operation, including fundamental concepts and educational features (Koehler et al., 2017). Such professional knowledge requires expertise in technological frameworks and models, as well as the principles and conceptual foundations of various innovations, and the rationale behind selecting particular technologies that enhance teacher effectiveness (Voogt and Mckenney, 2017). Educators who possess a firm theoretical grounding understand the advantages and restrictions of multiple technology instruments to select applicable integration plans that support teaching situations in diverse educational settings.

Practical knowledge emphasizes obtaining first-hand teaching skills and adequate technology mastery (Angeli and Valanides, 2009). Proceeding with professional efficiency means developing competencies essential for operating technology tools properly and making problem-solving adjustments based on student needs and educational targets within their learning environments. Through practical knowledge, teachers can effectively administer technological tools and find innovative solutions to integration issues, while diagnosing teaching issues and adjusting technology use based on instructional priorities (Chai et al., 2013). Practical technological expertise is acquired through direct experience and trial and error while operating technological tools within authentic educational environments (Angeli and Valanides, 2009).

According to the research literature, integrating educational technology requires a strong foundation in both theoretical and practical knowledge to achieve effective and sustainable practices. Teachers need a theoretical understanding to make informed decisions about tools and practical knowledge that builds their skills and confidence in using technology in classroom teaching (Lee et al., 2024; Ng et al., 2023; Shi, 2024). According to scholarly research, theoretical expertise alone cannot ensure the sustainable integration of technology, as teachers with insufficient practical skills will encounter challenges in this process. Teachers who master practical technology skills often face obstacles when attempting to integrate technological implementation with educational goals, as they lack a theoretical understanding (Koehler et al., 2017; Voogt and Mckenney, 2017). Education fosters the sustainable integration of technology, which requires teachers to strike a balance between their theoretical background knowledge and practical skills. Educational institutions should implement professional development initiatives that combine theoretical instruction on technology integration with hands-on application possibilities, allowing teachers to develop sustainable educational practices (Tondeur et al., 2012; Voogt and Mckenney, 2017).

*H1*: Theoretical AI knowledge positively affects teachers’ ability to integrate AI sustainably.

*H2*: Practical AI knowledge has a positive impact on teachers’ ability to integrate AI sustainably.

### 2.3 Teachers’ AI beliefs and attitudes

Teachers’ beliefs about artificial intelligence and their attitudes influence the classroom implementation of AI-related knowledge, as they decide whether to foster or hinder the incorporation of sustainable technology. Available studies show that teachers’ individual beliefs about utilizing modern technologies determine their readiness to use AI alongside other innovative tools for learning practices (Al Darayseh, 2023; Ayanwale et al., 2022; Ertmer and Ottenbreit-Leftwich, 2010; Teo et al., 2018). The positive attitudes teachers display toward AI lead them to become more motivated and open to experimenting with AI tools repeatedly, as they seek continuous improvement in their teaching methods, which in turn leads to better learning outcomes (Nazaretsky et al., 2022; Luo et al., 2025; Tondeur et al., 2017). The connection between positive beliefs and attitudes produces stronger self-efficacy, enabling teachers to develop confidence in using AI knowledge theoretically and practically (Tondeur et al., 2012; Yang and Chen, 2023).

Negative attitudes and apprehension, as well as skeptical beliefs about AI, prevent teachers from adopting AI technologies properly, even though they already possess sufficient theoretical expertise and practical experience (Ifenthaler and Schweinbenz, 2013). Instructors with false beliefs or doubts about AI applications in education tend to avoid technology adoption by remaining unconvinced about using AI teaching instruments (Hsu, 2016; Wang et al., 2023). Teachers’ fears about AI stem from doubts regarding its technical complexity, workload reliability, data protection, and concerns that educational professionals may lose their instructional responsibilities (Chiu and Chai, 2020; Teo, 2009). The school environment and professional development formats for teaching professionals need improvement to transform teacher perceptions and combat opposition against AI educational usage (Chai et al., 2013).

Research indicates that teachers’ beliefs and attitudes are crucial in integrating their knowledge of AI with their ability to utilize it effectively in classrooms. Teachers with advanced education and practical experience in AI technology cannot effectively integrate AI-based instruction into their lessons unless they adopt a favorable perspective and a belief in the educational value of AI (Ertmer and Ottenbreit-Leftwich, 2010; Luo et al., 2025). Teachers must combine a correct understanding of AI with a supportive mental stance and educational expertise to develop meaningful long-term use of AI-based educational tools (Hsu, 2016; Teo et al., 2018). Organizations responsible for educational management must base their work on belief development and attitude transformation because perceived self-efficacy is combined with AI acceptance beliefs (Luo et al., 2025).

*H3*: Teachers’ beliefs and attitudes toward AI positively affect sustainable AI integration ability.

*H4*: Teachers’ beliefs and attitudes toward AI positively mediate the relationship between theoretical knowledge and the ability to integrate AI sustainably.

*H5*: Teachers’ beliefs and attitudes toward AI positively mediate the relationship between practical knowledge and the ability to integrate AI sustainably.

## 3 Methodology

This study specifically focuses on primary school teachers, and the Near East University ethical committee granted ethical permission for this study (EB/1120). The motivation for choosing primary school teachers, and not higher educational levels, was due to the limited focus on AI in primary education. More precisely, most reviewed studies were related to AI in higher education, while some were related to AI in K-12 education. In contrast, less than 3% of the studies focused on AI in primary education. Additionally, a limited number of recent publications have focused on the education of children with AI-based learning (Alam, 2022; Ali et al., 2019; Ha and Lee, 2019; Leifler, 2020; Su and Yang, 2022, 2023). As indicated by UNICEF, primary education constitutes the foundation of development, where children acquire essential skills that equip them for life, employment, and active citizenship. Quality education empowers children and adolescents, protects their health and well-being, and breaks the cycle of poverty. It also enables nations to foster economic growth and social unity (UNICEF, 2024). Therefore, the scope of this study is built explicitly on primary education.

This research employs an analytical, quantitative approach to investigate the relationships between AI knowledge, beliefs, and attitudes, as well as AI integration among primary school teachers, with the aim of facilitating the sustainable implementation of AI tools into their curriculum. For the data collection of this study, a scale developed and introduced by Ferikoğlu and Akgün (2022), titled the Teachers’ Artificial Intelligence Awareness Scale (Ferikoğlu and Akgün, 2022), was used with the authors’ permission. This scale was created to assess teachers’ level of awareness regarding AI in education and their inclinations toward the notion of AI and its sub-branches, with a proven reliability (Cronbach’s alpha score of 0.986). By its authors, the scale was created in Turkish and was assessed by field specialists for its clarity and language intelligibility. In addition to some demographical questions such as age, educational background and experience, the scale consists of 51 items that is prepared as a 5-point Likert type survey (1: “strongly disagree,” 2: “disagree,” 3: “not sure,” 4: “agree” and 5: “strongly agree”) which investigates the following four dimensions: Theoretical Knowledge, Practical Knowledge, Beliefs and Attitudes and Sustainable AI Integration Ability. 11 of the scale measure teachers’ theoretical knowledge about AI; 16 of the items measure teachers’ practical knowledge of AI; 14 items measure teachers’ beliefs-attitudes toward AI; 10 items measure teachers’ sustainable integration ability (Ferikoğlu and Akgün, 2022). The factors and items from Ferikoğlu and Akgün's (2022) questionnaire have been validated through confirmatory factor analysis.

340 primary school teachers from Turkish Cypriot regions across Cyprus participated in the study using a convenience sampling methodology. The investigation used data derived from Krejcie and Morgan (1970) to determine the final sample number (Krejcie and Morgan, 1970). Across the representative sample, various educational institutions and teachers from distinct professional levels participated. Schools were classified by age groups, institutional type (public vs. private), and the number of years their teachers had professional experience. The researchers conducted this classification to achieve complete representation. By applying this method, researchers included a range of primary education environments throughout Cyprus. To address potential common method bias, we conducted Harman’s single-factor test. The results showed that no single factor accounted for more than 40% of the variance, indicating that common method variance was not a serious concern (Wang et al., 2024).

Both online and paper-and-pencil versions of a structured questionnaire were used to collect data, as they offered accessibility benefits and provided teachers with quick response rates. The questionnaire comprised five primary sections: demographic information, practical AI knowledge, theoretical AI knowledge, and teachers’ beliefs and attitudes toward AI and sustainable AI integration ability in teaching. The demographic section (Table 1) provides essential background details about the participating teachers. In the AI usage section, teachers were asked to indicate the frequency and types of AI-based educational tools they utilized in their classrooms, such as intelligent tutoring systems, adaptive learning platforms, and automated assessment applications.

**Table 1:** Demographic statistics of the participants.

Age group	Experience	Educational background	School type
20–29: 77 (23%)	10 and less: 132 (39%)	Bachelor’s: 226 (67%)	Public: 200 (59%)
30–39: 149 (44%)	10–19: 118 (35%)	Master’s: 69 (20%)	Private: 140 (41%)
40–49: 62 (18%)	20 and over: 90 (26%)	PhD: 5 (1%)	
50 and over: 52 (15%)		Academy: 40 (12%)	

The demographic profile of the teachers who participated in this study reflects a diverse range of age groups, educational backgrounds, teaching experience, and school types. Most teachers fell within the 30–39 age group (43%, *n* = 149), followed by younger teachers aged 20–29 (22%, *n* = 77). Teachers aged 40–59 represented 18% (*n* = 62) of the respondents, and those aged 50 or older comprised 15% (*n* = 52) of the sample. Regarding educational background, most teachers held a bachelor’s degree (66%, *n* = 226), while 20% (*n* = 69) held a master’s degree. A smaller proportion had academy-level qualifications (11%, *n* = 40), and only a few participants (1%, *n* = 5) held PhDs. The respondents also had varied professional experiences. Teachers with less than 10 years of teaching experience formed the largest group (38%, *n* = 132), followed closely by those with 10–19 years of experience (34%, *n* = 118). Teachers who had taught for 20 years or more accounted for approximately one-quarter of the sample (26%, *n* = 90). Lastly, the teachers were from public and private schools, with public school teachers making up the majority of respondents (58%, *n* = 200), while teachers from private institutions represented a substantial portion (41%, *n* = 140). This variety gave comprehensive insights into AI usage and attitudes across educational contexts.

## 4 Results

The research explored the relationships between theoretical and practical knowledge of AI, beliefs and attitudes toward AI, and sustainable AI integration, using Structural Equation Modeling as the analytical method. SEM was chosen because it allows researchers to analyze observed variables in relation to latent variables, which enables them to validate the role of technological readiness in mediation effects. The questionnaire’s measurement scales underwent Confirmatory Factor Analysis (CFA) evaluation to validate their reliability and inspect their validity before SEM execution. The results of the CFA are presented in Table 2 below.

**Table 2:** Correlation matrix.

	Theoretical knowledge	Practical knowledge	Beliefs and attitudes	Sustainable AI-integration ability
Theoretical knowledge	0.74			
Practical knowledge	0.64***	0.78		
Beliefs and attitudes	0.72***	0.722***	0.90	
Sustainable AI-integration ability	0.61***	0.70***	0.85***	0.77

***p < 0.001.

The SEM analysis required two fundamental stages. The measurement model was evaluated regarding scale adequacy and validity using the standard CFI, TLI, and RMSEA goodness-of-fit indices. The AI integration assessment was followed by validation of the measurement model, after which researchers investigated the direct relationships between sustainable AI integration and the intermediary role of teachers’ beliefs and attitudes. The analysis employed bootstrapping to determine mediating effects and generate indirect effect concepts, along with their standardized confidence intervals.

Table 2 below provides insights into how various aspects of teachers’ knowledge and attitudes are related to their use of AI in teaching. The findings suggest that teachers with more positive beliefs and attitudes toward AI tend to integrate AI more effectively into their classrooms (*r* = 0.85, *p* < 0.01). This highlights the importance of addressing teachers’ attitudes to promote the successful integration of AI. The relationship between teachers’ AI practical expertise and their educational beliefs (*r* = 0.722, *p* < 0.01) and their ability to integrate AI tools (*r* = 0.70, *p* < 0.01) emerged as strong correlations. Teachers’ theoretical AI knowledge shows strong relationships with their practical skills (*r* = 0.64, *p* < 0.01) alongside their beliefs (*r* = 0.72, *p* < 0.01) and attitudes about AI (*r* = 0.72, *p* < 0.01). Teachers’ theoretical comprehension of AI directly affects teachers’ ability to sustainably integrate of AI technologies in educational instruction (*r* = 0.61, *p* < 0.01). The integration of sustainable AI practice by teachers requires developing theoretical knowledge and practical expertise through positive attitude enhancement programs.

All factor loadings for the measured factors were statistically significant and exceeded the threshold of 0.60 (see Table 3). As shown in Table 3, the composite reliability values for all constructs were above 0.70, while the average variance extracted (AVE) for each construct surpassed the recommended 0.50 threshold. These results confirm that the research measurements exhibit strong instrument reliability and convergent validity, which aligns with Fornell and Larcker’s (1981) criteria. Furthermore, discriminant validity was assessed to ensure that each construct was distinct and that items were more strongly associated with their respective constructs than with others. For each construct, the square root of the average variance extracted from values, as shown on the diagonal in the correlation matrix, exceeded the correlation coefficients between the constructs. This suggests good discriminant validity.

**Table 3:** Item factor loadings.

Factors	Factor loading	Significance	Mean	Composite reliability
Theoretical knowledge			3.89	0.90
Item 2 (Deep neural networks have been developed in the software world to mimic the function of the brain and nervous system)	0.72	Sig.		
Item 3 (Machines and programs are endowed with understanding and problem-solving abilities using machine learning or deep neural network methods with large amounts of data)	0.70	Sig.		
Item 4 (Artificial intelligence technologies process data to derive meanings and generate suggestions)	0.64	Sig.		
Item 10 (Machine learning refers to systems that can compare new data with existing data and identify patterns of similarity and difference between them)	0.61	Sig.		
Practical knowledge			3.56	0.90
Item 17 (Artificial intelligence helps better understand students’ individual needs)	0.74	Sig.		
Item 18 (Artificial intelligence helps to provide the highest quality education to children around the world in a personalized manner)	0.60	Sig.		
Item 19 (Artificial intelligence systems reduce the risk of errors in education)	0.73	Sig.		
Item 20 (Artificial intelligence systems accurately identify students’ personalities, strengths, and weaknesses)	0.72	Sig.		
Item 23 (Artificial intelligence technologies will support learning and career transitions)	0.60	Sig.		
Item 27 (With the use of artificial intelligence in lessons, classroom problems can be resolved)	0.60	Sig.		
Beliefs and attitudes			3.62	0.85
Item 30 (Using artificial intelligence in lessons increases efficiency)	0.66	Sig.		
Item 32 (Some artificial intelligence systems measure emotional reactions)	0.61	Sig.		
Item 34 (Artificial intelligence saves human lives)	0.63	Sig.		
Item 35 (Investments in natural language processing libraries contribute to the advancement of artificial intelligence)	0.61	Sig.		
Sustainable AI-integration ability			3.61	0.90
Item 42 (With the use of artificial intelligence in lessons, classroom problems are addressed and resolved)	0.75	Sig.		
Item 45 (Many decisions are entrusted to machine learning-based algorithms)	0.72	Sig.		

Table 3 presents numerical data on construct measurements in the study, including factor loading results with statistical significance, mean scores, and composite reliability scores. The analysis reveals that all statistical factors exceed 0.60, indicating a strong correspondence between items and their corresponding constructs. Theoretical Knowledge demonstrates excellent internal consistency, as evidenced by factor loadings ranging from 0.70 to 0.80, an average score of 3.89, and a composite reliability (CR) of 0.90. Practical Knowledge obtained robust loadings exceeding 0.70, up to 0.92, revealing a mean score of 3.56, while showing a Cronbach’s alpha (CR) of 0.82 to verify its construct reliability. One item measuring Beliefs-Attitudes displays a 0.90-factor loading, with a mean score of 3.62, and a composite reliability of 0.85, confirming its strong measurement quality. The AI-Integration construct utilizes two measurement items, demonstrating satisfactory factor loadings of 0.71 and 0.83. The established internal reliability for this construct remains strong, as evidenced by its mean score of 3.61 and Cronbach’s alpha (α) of 0.90. Our results establish the reliability of the measurement model, as each construct demonstrates robust factor loadings and adequate composite reliability values, thereby ensuring the validity of the research measurement design. Furthermore, the model fit analysis results indicate that the measurement model demonstrates an acceptable fit, as evidenced by multiple indices of goodness of fit. The Comparative Fit Index (CFI) and Tucker-Lewis Index (TLI) both equal 0.90, approaching the recommended threshold of 0.80, which suggests a strong comparative fit. The Root Mean Squared Error of Approximation (RMSEA) = 0.05 falls within the acceptable range of ≤ 0.06, indicating a reasonable approximation of the model to the population. Additionally, the Standardized Root Mean Square Residual (SRMR) = 0.07 meets the recommended cut-off of ≤ 0.08, further supporting the adequacy of the model.

**Figure 1:**
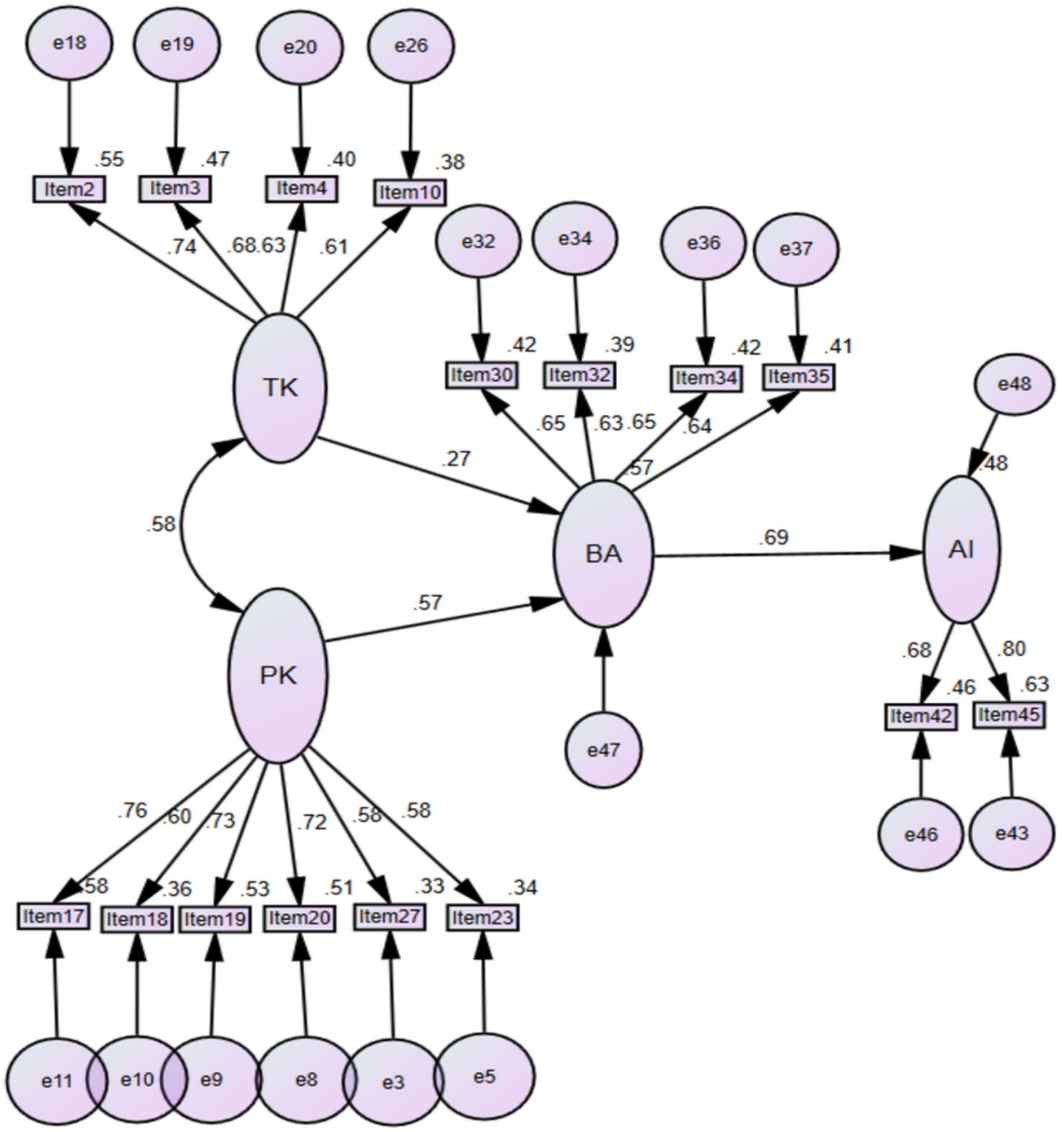
SEM path analysis.

Figure 1 shows the results of the path analysis, which was conducted using AMOS v21.

Table 4 presents the Structural Equation Modeling (SEM) analysis using the bootstrapping technique, which demonstrates that beliefs and attitudes serve as fundamental elements in teachers’ adoption of AI. Teachers’ beliefs and attitudes are most strongly influenced by theoretical knowledge (β = 0.57, *p* < 0.01). Beliefs and attitudes have a significant impact on the development of sustainable AI integration ability (β = 0.69, *p* > 0.01). The sustainable AI integration ability is explained by 48% by the model’s variables. The beliefs and attitudes showed an R-squared value of 58% which indicates that practical and theoretical knowledge explains 58% of the variance in beliefs and attitudes.

**Table 4:** Results of the structural equation modeling (SEM).

Hypothesis	Paths	Coefficients	Lower	Upper	*p*-value	S. E	C. R	*R* ^2^
H1	Theoretical knowledge → Sustainable AI-integration ability	0.48***	0.34	0.42	0.004	0.14	5.11	0.48
H2	Practical knowledge → Sustainable AI-integration ability	0.05	−0.12	0.21	0.56	0.14	0.62	
H3	Beliefs and attitudes → Sustainable AI-integration ability	0.69**	0.62	0.77	0.01	0.12	8.30	
H4	Theoretical knowledge → Beliefs and attitudes → Sustainable AI-integration ability	0.24**	0.13	0.41	0.01			
H5	Practical knowledge → Beliefs and attitudes → Sustainable AI-integration ability	0.58**	0.42	0.78	0.01			
	Theoretical knowledge → Beliefs and attitudes	0.27**	0.12	0.40	0.01	0.07	3.39	0.58
	Practical knowledge → Beliefs and attitudes	0.57**	0.45	0.72	0.01	0.09	6.12	

***p < 0.001; **p < 0.01.

Both theoretical and practical knowledge indirectly affect teachers’ ability to sustainably integrate AI, with teachers’ beliefs and attitudes serving as the mediating factor (β = 0.24, *p* < 0.001 and β = 0.58, p < 0.01, respectively). Research evidence suggests that educational knowledge about AI is insufficient to drive adoption, necessitating the development of positive user perceptions and confidence in AI applications. Educational institutions must teach their trainees basic AI skills and techniques, along with approaches to build favorable mindsets and tackle challenges that could arise from AI usage within schools.

## 5 Discussion

The research examined how primary teachers’ knowledge levels about AI affect their ability to sustainably integrate AI tools into their classroom practices, with teacher beliefs and professional attitudes being essential intermediary factors. The study provides essential data on how different knowledge levels and psychological variables influence the sustained deployment of educational technology in schools.

### 5.1 Theoretical implications

Results showed that teachers with a deep understanding of AI theory hold more positive beliefs and attitudes toward AI. According to previous research, theoretical knowledge is a fundamental requirement for teachers to understand both the advantages and educational applications of AI (Koehler et al., 2017; Voogt and Mckenney, 2017). Although theoretical knowledge has a strong influence on beliefs and attitudes, research has shown that direct theoretical knowledge does not independently predict AI integration. Knowledge is a necessary but insufficient condition for classroom integration since teachers require positive attitudinal mediation (Luo et al., 2025; Yang and Chen, 2023). The study findings indicated that practical AI knowledge failed to establish a link between any changes in teachers’ beliefs and attitudes and their actual ability to integrate AI sustainably. Teachers’ exposure to AI technology practices seems to defeat expectations that it automatically builds their comfort level and willingness to sustain technology integration. Focused training programs should build positive attitudes, self-efficacy, and confidence in AI while providing essential skills (Ertmer and Ottenbreit-Leftwich, 2010; Lee et al., 2024). Research findings suggest that theoretical knowledge has a substantial impact on how teachers develop a positive attitude toward artificial intelligence. The Structural Equation Modeling (SEM) analysis demonstrates that theoretical knowledge has a positive influence on teachers’ beliefs and attitudes, with a coefficient of β = 0.70 (*p* < 0.001). The findings of this analysis confirm previous research regarding theoretical knowledge in educational technology integration (Koehler et al., 2017; Voogt and Mckenney, 2017). The theoretical framework that teachers construct enables them to recognize the value and potential of AI technology, creating a space for positive attitudes toward AI applicability in educational contexts.

Educational and interpersonal acceptance of technology is a critical gateway for teachers to use and implement its practical use. The integration of AI among teachers depended heavily on theoretical and practical knowledge, as these factors significantly influenced their beliefs and attitudes (β = 0.36, *p* < 0.001; β = 0.10, *p* < 0.01, respectively). According to Tondeur et al. (2017) and Voogt and McKenney (2017), teachers require comprehensive training that combines psychological support and attitude development with technological understanding and practical skills (Tondeur et al., 2017; Voogt and Mckenney, 2017). Teachers’ beliefs and attitudes were identified as the key variables in this study. The study reveals that those psychological elements function as the most influential variables in determining the sustainable integration of AI. Teachers tend to have a positive perception of AI as applicable, exhibiting self-confidence in managing AI tools, which leads them to implement these tools sustainably in their teaching practices. Research in educational technology demonstrates that teacher attitudes and perceived ease of use positively impact the adoption of educational technology (Al Darayseh, 2023; Chounta et al., 2022; Teo et al., 2018; Tondeur et al., 2017). The implementation of educational policy has clear, practical indications for educational policymakers and educational institutions (Knox, 2020). Teachers require more than traditional information sharing to successfully integrate AI into training programs, as specific methods must be employed to foster positive attitudes and self-efficacy skills (Ayanwale et al., 2022; Ng et al., 2023). Policies that include workshops, mentoring sessions, and continuous support communities help teachers manage their stress and develop technical proficiency to establish lasting AI integrations in the educational system.

### 5.2 Practical implications

According to the findings, teacher preparation programs need to move beyond teaching technical skills alone. Educational stakeholders who lead teaching organizations and training programs should develop professional learning approaches that foster both conceptual understanding and motivational competencies in teachers simultaneously. Teacher preparation programs that deliver integrated teachings of AI theory and practice will help educators develop knowledge about the pedagogical advantages of AI while overcoming doubts about its use in education. Research findings indicate that encouraging educational settings are essential because they foster positive attitudes and beliefs in students, promoting learning. Combining institutional backing with straightforward communication about AI and chances to work with colleagues and real-time AI technology contact will reinforce favorable views among teaching staff. A positive environment makes it easier for teachers to overcome their fears about technological complexity, data security, and role changes in the workplace because these issues are known barriers to long-lasting technology integration (Chiu and Chai, 2020; Cojean and Martin, 2022; Ifenthaler and Schweinbenz, 2013).

### 5.3 Limitations and future research

The diverse participant population enhances the generalizability of the research findings across different populations. These results apply to various teaching situations, given the diverse participant backgrounds, including age diversity, educational training, and length of teaching experience. Future investigations should analyze institution-created policies with technological resources, professional support strategies, and cultural perceptions of technology to enhance knowledge about how these variables affect the relationships discovered in this study.

## 6 Conclusion

The research makes a substantial contribution to educational technology research by demonstrating that teachers’ beliefs significantly influence the connection between AI knowledge and the teachers’ ability to integrate AI practices sustainably. Theoretical knowledge of AI influences teachers’ beliefs and attitudes, leading to their more effective integration of sustainable AI tools in primary education. The achievement of successful integration depends on both theoretical and practical knowledge; however, practical training alone is insufficient. Teacher training must utilize a comprehensive educational system that integrates theoretical instruction with hands-on practice and psychological support to foster favorable attitudes and beliefs. Educational policymakers and institutions must develop unified, holistic strategies that focus on both mental performance and emotional development, as this ensures success in integrating AI technology into students’ primary education.

## Data Availability

The raw data supporting the conclusions of this article will be made available by the authors, without undue reservation.
